# Hirayama's Disease: About a Clinical Observation

**DOI:** 10.7759/cureus.75445

**Published:** 2024-12-10

**Authors:** Boutayna Touiti, Zouhayr Souirti

**Affiliations:** 1 Neurology, Hassan II University Hospital, Fez, MAR

**Keywords:** amyotrophy, cervical myelopathy, dynamic flexion mri, hirayama's disease, young adults

## Abstract

Hirayama disease, also known as non-progressive juvenile spinal muscular atrophy of the upper limbs, brachial monomelic amyotrophy, or benign focal atrophy, affects the C7 D1 myotomes; an electromyogram (EMG) shows neurogenic damage in the C7-C8-T1 territories. It causes weakness and amyotrophy of the distal upper limb. Although it usually occurs on one side only, bilateral symmetric cases of Hirayama disease have occasionally been described. It is a slow, progressive disease, and its evolution can have a good prognosis when detected early in the process. We describe a clinical observation of Hirayama disease, including its clinical and paraclinical peculiarities, and compare it to data from previous studies.

## Introduction

Hirayama disease is a benign cervical myelopathy that was discovered by Hirayama in 1959 [1]. Research has confirmed this condition as a distinct disorder separate from motor neuron disease; it arises from spinal cord compression by the posterior dural sac during neck flexion [1]. It mainly affects young males and is clinically characterized by progressive weakening and atrophy of the muscles in the hands and forearms. The atrophy is unilateral in most patients, bilateral and asymmetrical in a few, and rarely symmetrical [2]. This article describes our case of Hirayama disease and its clinical and paraclinical peculiarities.

## Case presentation

**Figure 1:**
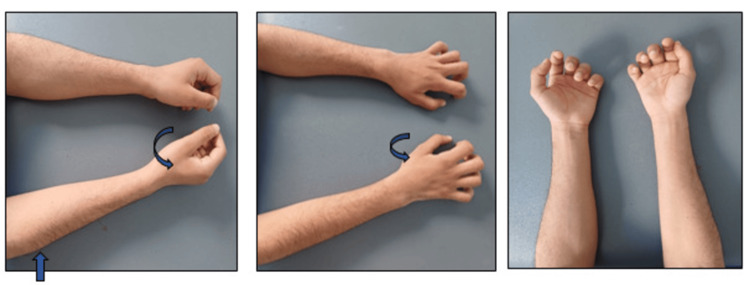
Photograph of our patient Note the atrophy of the first right dorsal interosseous (curved arrow) and other small muscles of the hand, which have a claw-like appearance, and the preservation of the brachioradial muscles (right arrow), which gives the appearance of oblique muscular atrophy.

A 19-year-old male student presented to our neurology consultation with a progressively developing weakness in his right hand. He had no medical history, and the family history revealed no similar cases. The patient reported fasciculations preceding the onset of the deficit several months ago. Clinical examination found a deficit and amyotrophy of the first right dorsal interosseous and other small muscles of the right hand, with a claw-like appearance and preservation of the brachioradial muscles (Figure 1). Moreover, the rest of the neurological examination was unremarkable.

We noted electrophysiological abnormalities: a reduction of ulnar compound muscle action potential (CMAP) and the abolition of the F wave of the right ulnar and median nerves (Table 1). Sensory conduction studies were normal (Table 2). Needle electromyography (EMG) found neurogenic changes in the C7 and C8 distribution with sparing of the brachioradialis muscle on the right side (Table 3). There was no effect on the lower limb muscles.

**Table 1:** Motor conduction study "R" stands for right, and "L" stands for left.

Nerve	Location	Latency (ms)	Amplitude (mv)	Area (mV.ms)	Conduction Velocity (m/s)
Median R	Wrist	3.7	10.7	47.6	-
	Elbow fold	8.0	10.5	46.5	58.4
	Axillary hollow	9.3	10.7	47.0	74.2
Ulnar R	Wrist	3.9	2.0	7.2	
	Below elbow	7.9	2.2	8.6	66.2
	Above elbow	9.3	2.0	7.4	57.1
Median L	Wrist	3.8	10.1	46.6	
	Elbow fold	8.0	9.6	45.6	59.5
	Axillary hollow	9.6	9.1	45.0	69.1
Ulnar L G	Wrist	3.0	7.5	32.8	
	Below elbow	7.6	6.4	30.0	55.3
	Above elbow	8.7	6.5	30.8	71.4
Radial D	Elbow fold	3.4	4.6	21.6	
	Shoulder	5.7	4.2	22.8	70.2

**Table 2:** Sensory conduction study "R" stands for right, and "L" stands for left. "Antidromic" refers to the direction of the nerve conduction opposite to the normal physiological direction.

Nerve	Latency (ms)	Amplitude (µV)	Area (mV.ms)	Conduction velocity (µV)
Radial R	1.5	50.9	36.2	60.8
Median palm R	1.3	54	25.3	72.0
Median palm L	1.2	51.8	23.6	70.8
Ulnar Antidromic R	2.4	15.5	9.6	50.0
Ulnar Antidromic L	2.3	12.0	7.8	53.9

**Table 3:** The F wave

Test	Latency (ms)
Abd 5th Finger, Ulnar, C8 T1, Right	23.0
Abd 5th Finger, Ulnar, C8 T1, Left	29.8
Short Abductor of the 1st Finger, Median, Right	23.0
Short Abductor of the 1st Finger, Median, Left	29.9

**Figure 2:**
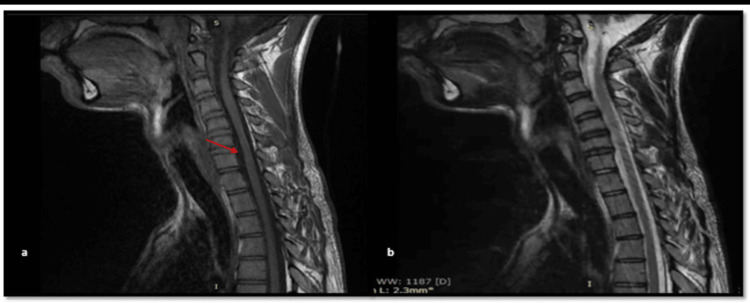
Cervical MRI Sagittal-T1 sequence (a) and sagittal-T2 (b) show cervical spine straightness and moderate atrophy at the C6-C7 level, with enlargement of the subarachnoid space (arrow) without an intramedullary hypersignal.

Our patient had a cervical MRI in the neutral position, which revealed straightening of the cervical spine without intramedullary hyperintensity (Figure 2). Due to unavailability, we did not perform a cervical MRI in flexion. Based on clinical criteria, EMG, and cervical MRI, the diagnosis of Hirayama disease was made.

Treatment was recommended for our patient; symptomatic management, including analgesics and anti-inflammatory medications, was prescribed to manage any associated pain; physiotherapy focused on maintaining muscle strength and preventing atrophy in unaffected muscle groups with a cervical collar. The course was marked by clinical stability.

## Discussion

Hirayama disease (HD) is a rare, juvenile cervical flexion myelopathy mainly affecting young males. It is responsible for a purely motor distal involvement of the upper limbs, with a slowly progressive evolution in the territory of the C7 to T1 metameres [3]. The physiopathology of the disease is subject to debate and remains unclear. However, findings suggest a disproportionate growth of the vertebral column compared to the dural sac, causing anterior displacement and compression of this cervical cord and resulting in atrophic changes in the anterior horn [3,4]. HD follows a biphasic evolution, with a progressive worsening of symptoms over three to four years, followed by a phase of disease stabilization [5]. Bilateral and symmetrical involvement are rare conditions that may be considered a severe form of Hirayama disease [3,6,7].

The diagnosis of HD is determined through clinical examination, EMG, but especially on cervical MRI in neutral or flexed on cervical MRI in a neutral or flexed position (Table 4). The typical results of cervical MRI in a neutral position and conventional imaging are listed in Table 4. During cervical MRI in flexion, an anterior migration of the lower cervical cord and the posterior dural sac is observed associated with an enlargement of the posterior epidural space in which dilated veins appear. The recommended range of cervical flexion is between 30 and 40 degrees. The flattening of the spinal cord in a flexed position is inversely correlated with the duration of disease development [8]. The differential diagnosis of HD must exclude syringomyelia, amyotrophic lateral sclerosis, cervical myelopathy, spinal tumor, and traumatic myelopathy [2].

**Table 4:** Diagnostic criteria for Hirayama's disease Adapted from [2] EMG: electromyography

Clinical manifestation	Onset in adolescence, weakness and atrophy of the distal part of the upper limb, sparing of the brachioradial muscle (long supinator), irregular, coarse tremor in the fingers of the hand, absence of clear sensory deficit, abnormalities of reflexes and cranial nerves; no evolution and progression a few years after the onset
EMG	Chronic clinical or subclinical denervation in the affected muscles
Cervical MRI in a neutral position	Localized atrophy of the lower cervical cord, abnormal curvatures, asymmetrical flattening of the medulla, pear-shaped medulla in the cross-section, parenchymal changes in the lower cervical cord: intramedullary hypersignal
Dynamic cervical MRI in flexion	Anterior migration of the dura mater in flexion with a large epidural space and enhancement of the epidural component

The treatment of Hirayama disease is generally limited; there have been isolated reports showing a beneficial effect of wearing a cervical collar or undergoing physiotherapy [9]. Limiting neck flexion using a cervical collar may help prevent disease progression [6]. Surgical intervention has been reported to be beneficial in treating Hirayama disease and improving hand function [10].

## Conclusions

Hirayama disease is a rare condition that presents challenges in both positive and differential diagnoses. It is important to know about this disease because it has a benign course, making it easy to confuse with other anterior horn disorders that have a poor prognosis, in particular amyotrophic lateral sclerosis.
